# Runners Employ Different Strategies to Cope With Increased Speeds Based on Their Initial Strike Patterns

**DOI:** 10.3389/fphys.2021.686259

**Published:** 2021-11-02

**Authors:** Antonis Ekizos, Alessandro Santuz, Adamantios Arampatzis

**Affiliations:** ^1^Department of Training and Movement Sciences, Humboldt-Universität zu Berlin, Berlin, Germany; ^2^Berlin School of Movement Science, Humboldt-Universität zu Berlin, Berlin, Germany; ^3^Olympic Training Center, Berlin, Germany

**Keywords:** strike index, human locomotion, running economy (RE), velocity, running strategy, foot strike patterns

## Abstract

In this paper we examined how runners with different initial foot strike pattern (FSP) develop their pattern over increasing speeds. The foot strike index (FSI) of 47 runners [66% initially rearfoot strikers (RFS)] was measured in six speeds (2.5–5.0 ms^−1^), with the hypotheses that the FSI would increase (i.e., move toward the fore of the foot) in RFS strikers, but remain similar in mid- or forefoot strikers (MFS) runners. The majority of runners (77%) maintained their original FSP by increasing speed. However, we detected a significant (16.8%) decrease in the FSI in the MFS group as a function of running speed, showing changes in the running strategy, despite the absence of a shift from one FSP to another. Further, while both groups showed a decrease in contact times, we found a group by speed interaction (*p* < 0.001) and specifically that this decrease was lower in the MFS group with increasing running speeds. This could have implications in the metabolic energy consumption for MFS-runners, typically measured at low speeds for the assessment of running economy.

## Introduction

Foot strike patterns (FSP) describe the location of the first contact area of the foot with the ground (Cavanagh and Lafortune, 1980) during running. At comfortable speeds, runners most commonly strike with the rear part of the foot (∼78%), while the rest strike with the middle or the front part of the foot (Santuz et al., 2016). The two strategies provide very distinct running patterns, exhibiting differences in a plethora of biomechanical characteristics (Hayes and Caplan, 2012; de Almeida et al., 2014; Almeida et al., 2015; Strauts et al., 2015; Valenzuela et al., 2015). For instance, it is well accepted that runners that strike the ground with the heel exhibit a lower peak vertical ground reaction force, lower external dorsiflexion moment and range of motion, while having a higher loading rate of the vertical ground reaction forces and knee extension moment in comparison to runners with a more anterior point of force application (Almeida et al., 2015; Valenzuela et al., 2015). Moreover, certain FSPs have been linked to different injuries (Cheung and Davis, 2011; Daoud et al., 2012; Rice et al., 2013) and to affect performance (Di Michele and Merni, 2014; Ogueta-Alday et al., 2014; Ekizos et al., 2018).

The common strategy employed by humans to increase speed until ∼7 ms^–1^ is by exerting larger vertical ground reaction forces (Arampatzis et al., 1999; Weyand et al., 2000), which leads to increments in step length (Mercer et al., 2002; Dorn et al., 2012). Ground reaction forces subsequently increase the loading on the human system and have to be produced in shorter contact times that are associated with increasing velocities (Gatesy and Biewener, 1991; Arampatzis et al., 2000). Except the overall higher loading, the transition from a lower to a higher speed taxes the human system with an increased oxygen consumption. However, humans maintain similar energy costs (J/kg per meter distance) in a range of running speeds (Margaria et al., 1963; Carrier et al., 1984; Bramble and Lieberman, 2004). From a mechanical point of view, it has been suggested that these increases in running speed are achieved through a repositioning of the foot in relation to the ground. It is suggested that runners gradually adapt their FSP in order to modify the impact of loading or energy costs (Cheung and Davis, 2011; Cheung and Rainbow, 2014; Di Michele and Merni, 2014) and gradually employ a more anterior point of force application at first contact (Keller et al., 1996). However, previous reports did not find a consistent behavior regarding the changes of FSP with increasing speeds. Some studies report that the point of force application moves to the anterior with increasing speeds (Keller et al., 1996; Wang et al., 2018), but this alteration was not confirmed by other studies (Breine et al., 2014, 2019; Cheung et al., 2017). Furthermore, Forrester and Townend (2015), using the foot strike angle (i.e., angle of the foot with respect to the ground in the sagittal plane) as assessment parameter to classify FSP, found that the most runners did not change their initial foot strike angle by increasing running speeds. However, they identified also a cluster of rearfoot strike (RFS) runners that showed a decrease in foot strike angle indicating a trend to midfoot strike (MFS) patterns at higher speeds (Forrester and Townend, 2015). It seems, therefore, that runners are using diverse strategies concerning the FSP behavior to cope with increasing speeds.

Until now, there is no established consensus regarding the changes in FSP with increasing speeds. FSP is a discrete rather than a continuous variable (Breine et al., 2014) and thus changes within a given strike pattern may not be considered examining only the possible transfer from RFS to MFS and vice versa. Thus, a numerical continuous parameter like the foot strike index (FSI), may be a more appropriate way to investigate the modulation of FSP in different running speed conditions (Breine et al., 2014; Santuz et al., 2016). At speeds, which can be sustained for longer periods of time, the human system is more comfortable to exhibit its preferential or more familiar FSP. When increasing speed, the system is forced to accommodate the higher loads and alterations in FSI may be associated with the runners FSP at the comfortable speed. Non-rearfoot strikers, for instance, have a lower margin to increase their FSI anteriorly compared to rearfoot runners. Consequently, runners with a non-rearfoot strike pattern may retain a similar FSI throughout increasing running speeds. It is therefore possible, that the strategies of rearfoot and non-rearfoot strike runners could develop differently as speed progresses and particularly an alteration of FSI toward anterior only in rearfoot runners could be expected. In the current study, we examined the effect of speed on the FSI separately for runners with a rear and non-rear foot strike pattern. We hypothesized (1) a change of FSI in runners with a rear strike pattern toward the fore of the foot, leading to a higher percentage of non-rear foot runners by increasing running speed and (2) runners with an initial non-rear strike pattern would maintain the same strike pattern strategy.

## Methods

### Experimental Design

In the current study 47 young adults who were recreational runners (37 males and 10 females, training sessions per week: 3.7 ± 1.6, training duration per week 5.1 ± 2.8 h) have been recruited (age: 27.8 ± 4.8 years, height: 177.3 ± 8.6 cm, mass 70.9 ± 9.2 kg). For each participant the measurement took place on a single day. None of the participants had any neuromuscular or musculoskeletal impairments at the time of the measurements. Moreover, in the 6 months prior to the day of the measurements, none of them have suffered any injury to the lower limbs. All participants gave informed consent and approval of ethics has been acquired from the appropriate committee of the Humboldt-Universität zu Berlin (HU-KSBF-EK_2018_0013).

For the measurements we used a treadmill (mercury, H-p-cosmos Sports & Medical GmbH, Nussdorf, Germany) with an integrated pressure plate operating at 120 Hz (FDM-THM-S, zebris Medical GmbH, Isny im Allgäu, Germany). After a self-selected warm-up, the participants ran shod at six predefined sub-maximal fixed velocities; 2.5, 3.0, 3.5, 4.0, 4.5, and 5.0 ms^−1^. The chosen speeds were comfortably attainable by all participants for small periods of time. While in non-homogeneous groups relative intensity can provide methodological advantages, the homogeneity presented in our cohort meant we could use fixed speeds. As such, possible differences in the relative intensity would not skew our results and comparability with other studies is increased. The duration of the run at each speed was 2 minutes, of which the first minute was used as familiarization to the specific speed and the latter minute was extracted for subsequent analysis.

To calculate the contact time of each step we used the pressure plate data from the treadmill. The time that each foot was in contact with the ground has been calculated based on the time difference, between the first non-zero data after the swing phase and the first zero in the pressure data right after toe-off. We used the average of all contact times of both feet in all steps per trial per person for the statistical analysis. Cadence was calculated from the number of steps detected over the whole trial period. Subsequently, step length, step time and flight time were calculated based on these values. The duty factor was calculated as the ratio of contact time over step time.

The FSP was numerically quantified using the pressure distributions from the instrumented treadmill, through the strike index. The FSI is defined as the distance from the heel to the center of pressure at first impact, relative to the total foot length and was calculated based on the recorded foot pressure distribution using a validated custom algorithm (Santuz et al., 2016). In short, after physically measuring the shoe length (to account for incomplete footstrikes), the algorithm compares it to the calculated length (i.e., using the pressure plate data) and corrects the footstrikes when necessary (Santuz et al., 2016). The first recorded data (i.e., initial contact) at touchdown of each foot in every step are then localized to the full length of the foot. The values, therefore, range from the most posterior part of the heel representing 0 up to the most anterior part of the toes representing 1 (non-dimensional). In our paper we aimed at showing the behavior of the system as a whole and thus we used the average of the strike indexes of both feet in all steps per trial per person. The symmetry in the FSI between left and right foot was quite high depicting an association of *R*^2^ = 0.887 and differences were not statistically significant (*p* > 0.05) in all investigated speeds.

Generally, footstrikes are divided in three distinct categories based on where the first impact is located, in relation to the whole foot. A RFS is considered one that provides a strike index lower than 0.33 and thus first touch occurs at the heel of the foot, a midfoot strike one that provides values between 0.33 and up to 0.66 (approximate point of the metatarsophalangeal joints), and a forefoot strike one with values above 0.66 (Cavanagh and Lafortune, 1980; Hasegawa et al., 2007). Due to forefoot strikers exhibiting a low prevalence in the general population (Hasegawa et al., 2007; Larson et al., 2011; Santuz et al., 2016), in this study the participants exhibiting a mid- or a forefoot strike have been grouped together as MFS for all further analysis.

### Statistics

We defined two groups based on the FSI at the slowest running speed (i.e., 2.5 ms^−1^). In that way a RFS (*n* = 31) and a MFS group (*n* = 16) have been identified. To further examine the differences and development of FSI, contact time, cadence, step length, step time, flight time and duty factor with speed based on the identified groups, we performed a two-way repeated measures ANOVA. Speed was selected as a 6-level within-subject factor and groups (RFS, MFS) as the between-subject factor. The level of significance was set to α = 0.05.

## Results

**FIGURE 1:**
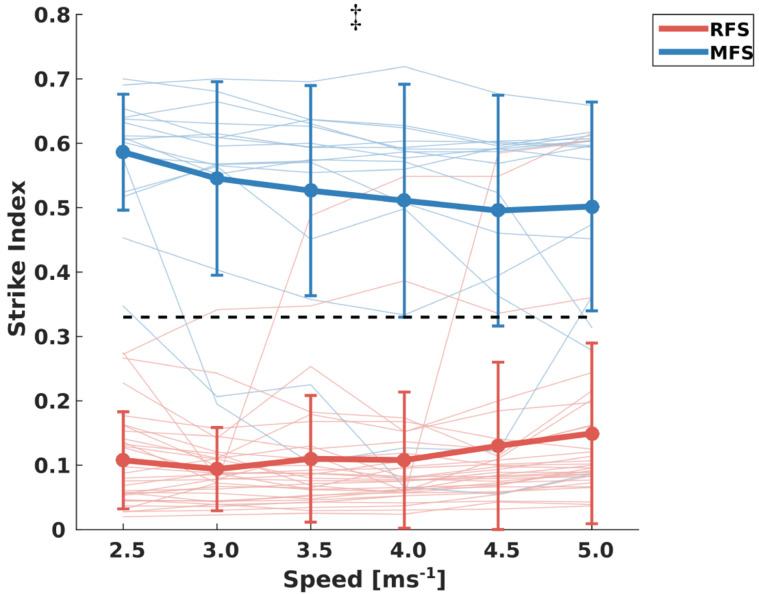
Mean ± standard deviation of the strike indexes throughout the examined speeds and the individual values for both rearfoot strikers (RFS) and mid-forefoot strikers (MFS). The black dotted line indicates the separation between RFS (below 0.33 of strike index) and MFS (above 0.33 of strike index). ‡: statistically significant group × speed interaction (*p* < 0.05) indicating a decrease of strike index only in MFS group.

Investigating the effect of running speed for the FSI we found an interaction between the two groups [*F*_(2,5)_ = 5.2, *p* = 0.005; Figure 1]. The *post hoc* analysis by means of a repeated measures ANOVA revealed a significant decrease in the FSI in the MFS group [*F*_(2,5)_ = 4.3, *p* = 0.018] and no significant differences in the RFS group [*F*_(1,5)_ = 2.5, *p* = 0.104].

**FIGURE 2:**
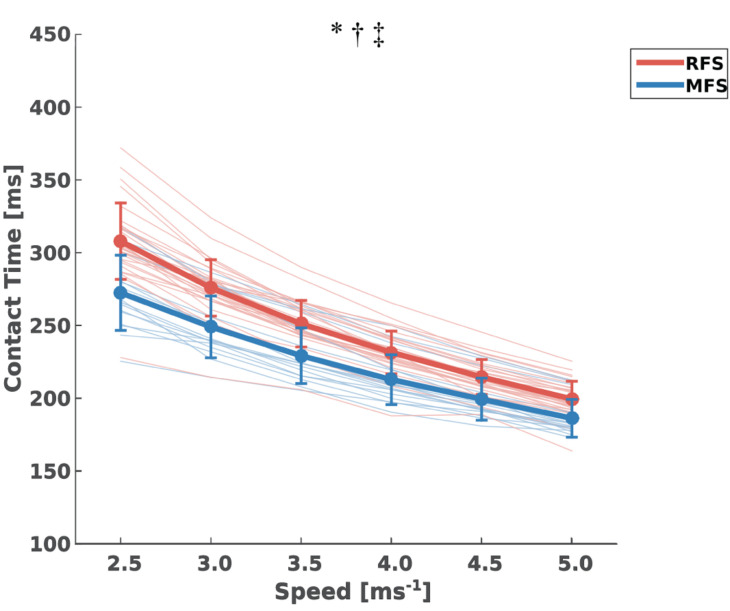
Mean ± standard deviation of contact times [ms] throughout the examined speeds and the individual values for both rearfoot strikers (RFS) and mid-forefoot strikers (MFS). *: statistically significant speed effect (*p* < 0.05); †: statistically significant group effect (*p* < 0.05); ‡: statistically significant group x speed interaction (*p* < 0.05) indicating a greater decrease of contact time in RFS group.

Contact times decreased significantly with increasing velocities [*F*_(2,5)_ = 799.8, *p* < 0.001] in both RFS and MFS groups, while between groups there was a significant effect [*F*_(1,5)_ = 8870, *p* < 0.001] with a clearly higher contact time in the RFS group (Figure 2). We found a group by speed interaction [*F*_(2,5)_ = 10.4, *p* < 0.001] in the contact time. In the *post hoc* analysis, both groups exhibited significantly decreased contact times with increasing velocities [RFS: *F*_(1,5)_ = 718.4, *p* < 0.001; MFS: *F*_(1,5)_ = 244.9, *p* < 0.001], therefore the interaction indicate a higher decrease of contact time in the RFS group.

Cadence increased with increasing speeds [*F*_(2,5)_ = 309, *p* < 0.001] and showed no interaction effects [*F*_(2,5)_ = 1.8, *p* = 0.180; Table 1]. Similarly, there was an increase [*F*_(2,5)_ = 2132, *p* < 0.001] and no interaction in the development of step length [*F*_(2,5)_ = 1.7, *p* = 0.197]. Step time decreased [*F*_(2,5)_ = 313, *p* < 0.001] as a function of running speed without any interaction effects [*F*_(2,5)_ = 1.9, *p* = 0.155]. Flight times increased significantly with increasing velocities [*F*_(2,5)_ = 138, *p* < 0.001] and showed a significant interaction between groups and speed [*F*_(2,5)_ = 4.2, *p* = 0.024]. *Post hoc* comparisons evidenced a statistically significant [*F*_(2,5)_ = 370, *p* < 0.001] lower flight time in RFS runners in all speeds (Table 1). Duty factor decreased significantly with increasing speeds and an interaction was also observed between speed and group [*F*_(2,5)_ = 4.9, *p* = 0.016]. In the *post hoc* comparisons RFS demonstrated a significantly greater [*F*_(2,5)_ = 7037, *p* < 0.001] duty factor compared to the MFS runners.

**TABLE 1:** Mean± standard deviation of the cadence, step length, step time, flight time, and duty factor for the rearfoot strikers (*n* = 31) and mid-forefoot strikers (*n* = 16).

**Speed [ms^−1^]**	**Cadence [steps/min]^∗^**			**Step length [m]^∗^**			**Step time [ms]^∗^**			**Flight time [ms]^∗†‡^**			**Duty Factor [%]^∗†‡^**		
	**RFS**	**MFS**	***p***	**RFS**	**MFS**	***p***	**RFS**	**MFS**	***p***	**RFS**	**MFS**	***p***	**RFS**	**MFS**	***p***
2.5	162 ± 9	164 ± 11	0.965	0.93 ± 0.05	0.92 ± 0.06	0.977	371 ± 21	368 ± 24	0.977	63 ± 30	96 ± 32	0.004	83.1 ± 7.5	74.2 ± 7.9	0.001
3.0	168 ± 11	168 ± 11	0.965	1.07 ± 0.07	1.07 ± 0.07	0.977	358 ± 24	358 ± 24	0.977	82 ± 25	109 ± 26	0.004	77.2 ± 5.9	69.8 ± 6.4	0.001
3.5	173 ± 12	174 ± 12	0.965	1.22 ± 0.08	1.22 ± 0.08	0.977	348 ± 24	347 ± 24	0.977	96 ± 23	118 ± 25	0.007	72.5 ± 5.3	66.2 ± 5.9	0.001
4.0	180 ± 12	180 ± 14	0.965	1.34 ± 0.10	1.34 ± 0.10	0.977	335 ± 24	335 ± 26	0.977	104 ± 22	122 ± 26	0.012	69.3 ± 4.9	63.8 ± 5.7	0.001
4.5	186 ± 12	184 ± 14	0.965	1.46 ± 0.10	1.48 ± 0.11	0.977	325 ± 22	329 ± 24	0.977	110 ± 20	129 ± 24	0.007	66.3 ± 4.3	60.9 ± 5.3	0.001
5.0	193 ± 13	190 ± 15	0.965	1.56 ± 0.11	1.59 ± 0.12	0.977	313 ± 22	318 ± 24	0.977	113 ± 19	132 ± 24	0.007	64.0 ± 4.2	58.8 ± 5.1	0.001

*Between groups comparisons through an independent samples Student’s *t*-test.*

*P-values are adjusted according to a Benjamini Hochberg false discovery rate analysis.*

*^∗^Statistically significant speed effect (*p* < 0.05).*

*^†^Statistically significant group effect (*p* < 0.05).*

*^‡^Statistically significant group × speed interaction (*p* < 0.05).*

## Discussion

In the current study, we examined the effect of speed on the FSI and contact time for RFS and MFS runners. Out of all 47 investigated participants, 31 (66%) were rearfoot striking at the initial examined speed (i.e., 2.5 ms^−1^) and 16 (34%) were mid- or forefoot striking. Only six participants (13%) exhibited a change in the FSP: three changed their FSP from RFS to MFS and three changed to a RFS while starting with a MFS. The rest of the participants did not change their initial FSP by increasing running speed. Further, we detected an overall decrease of FSI in the MFS group at higher running velocities. Both groups significantly decreased contact times with increasing speeds, however, the decrease was higher in the RFS group.

We hypothesized an increase of FSI in the RFS runners leading to a higher percentage of MFS runners by increasing running speed. Since only three participants changed from RFS to MFS our first hypothesis has been rejected. However, based on our use of the FSI, it was also shown that RFS runners do not alter the way their point of first contact within their chosen pattern either. This means they are maintaining a similar way of striking the ground throughout the examined speeds. In bipedal locomotion contact times decrease with increasing velocities (Gatesy and Biewener, 1991; Arampatzis et al., 2000) and RFS pattern is reported to have longer contact times than MFS (Hayes and Caplan, 2012; Di Michele and Merni, 2014; Ekizos et al., 2018). This could naturally lead participants that use RFS at lower velocities to change their strike pattern toward the fore of the foot. Dynamic stability during locomotion is a *sine qua non* concept and acute changes in the mechanics of running can cause instabilities in the system. In previous studies we found that acute changes in foot strike patterns (i.e., alteration from RFS to MFS) decrease the human dynamic stability during running (Ekizos et al., 2017, 2018). Maintenance of locomotor stability might be therefore a reason for the preservation of the foot strike patterns despite the increased running speed. Although contact time decreased significantly with the increased speed, the majority of the investigated RFS runners (87%) maintained the same FSP and minimized the changes in the FSI.

Midfoot strike runners also maintained their initial FSP throughout the examined speeds. However, in the MFS runners we found a significant (16.8%) decrease in the FSI with increasing speeds, which resulted in smaller differences in the FSI between RFS and MFS. This highlights that while the overall FSP did not change, the modification of the FSI within the MFS pattern indicates changes in the running strategy. At the same running speed, lower FSI is associated with a longer contact time (Gruber et al., 2013; Di Michele and Merni, 2014; Ekizos et al., 2018) and therefore the decrease of the contact time by increased speed was lower in MFS. The consequence was a reduction of the differences in the contact time between RFS and MFS runners by increasing speed. Both groups increased cadence and step length, and decreased step time in a similar way (Table 1). The flight time and consequently the duty factor on the other hand showed a different trend between groups indicating a greater time on the ground of the RFS runners by increasing speeds. There is evidence that the rate of metabolic energy consumption per body weight of running is inversely proportional to contact time (Kram and Taylor, 1990; Kram, 2000). Therefore, the lower decrease of contact time in MFS could affect the energy cost of running.

The higher FSI in the MFS group results in distinct distributions of the muscular output in the lower extremities between RFS and MFS runners [i.e., higher moments at the ankle and lower moments at the knee joint for MFS (Kulmala et al., 2013; Kuhman et al., 2016)], and leads to improvements in the cost coefficient (Ekizos et al., 2018). However, the improved cost coefficient due to the higher FSI in MFS did not improve running economy because of the lower contact time and thus greater rate of ground reaction force development (Ekizos et al., 2018). Traditionally, running economy is investigated in running speeds between 2.5 and 4 ms^−1^ (Heise and Martin, 2001; Arampatzis et al., 2006; Albracht and Arampatzis, 2013; Gruber et al., 2013; Craighead et al., 2014; Bohm et al., 2019) or as a percentage of the lactate threshold (Fletcher et al., 2009; Andersson et al., 2021) and several studies reported no differences in running economy between RFS and MFS (Gruber et al., 2013; Ekizos et al., 2018). In these speeds the average differences in the contact time between the investigated RFS and MFS were ∼9.6% and reduced to 6.5% in the 5.0 ms^−1^ speed and may decrease the negative effect of the contact time for running economy in MFS. Elite distance runners, for instance, who are commonly employing speeds >5.0 ms^–1^ (Hoogkamer et al., 2017) might have energetic benefits using MFS patterns. At least, our findings indicate that the investigation of running economy between RFS and MFS should be extended to higher running speeds.

Here, we found that most runners maintain their initial FSP with increasing running speed and that MFS runners even move the point of force application to the posterior. This means that until 5.0 ms^–1^ it is possible to increase the rate of force generation without a transition to a more anterior point of force application. However, based on our results we cannot answer how the FSI or other parameters will develop at speeds above 5.0 ms^–1^. Future investigations could improve our understanding concerning the effects of FSP on running mechanics and energetics during increased speeds, by considering measurements on metabolic energy consumption, lower leg kinetics and muscle mechanics (Arampatzis et al., 2006; Gruber et al., 2013; Bohm et al., 2019, 2021), as well as including runners who are accustomed with speeds higher than 5.0 ms^−1^.

## Conclusion

Although the majority of runners maintained their original FSP with increasing speed, we found that RFS and MFS runners employed different strategies to cope with this increase. Specifically, RFS runners maintained a similar FSI throughout the examined speeds, but MFS runners exhibited a significant reduction in the FSI, without this reduction being enough to change the FSP. Compared to RFS, the MFS group also decreased contact times slower with increasing speeds which could affect the measurement of the energy consumption in MFS runners, when this is measured only in slow speeds.

## Data Availability Statement

The raw data supporting the conclusions of this article will be made available by the authors, without undue reservation.

## Ethics Statement

The studies involving human participants were reviewed and approved by Humboldt-Universität zu Berlin. The patients/participants provided their written informed consent to participate in this study.

## Author Contributions

AE designed the study, carried out the experiments, and drafted the manuscript. AS designed the study, carried out the experiments, and edited the manuscript. AA designed the study and drafted the manuscript. All authors gave final approval for publication.

## Conflict of Interest

The authors declare that the research was conducted in the absence of any commercial or financial relationships that could be construed as a potential conflict of interest.

## Publisher’s Note

All claims expressed in this article are solely those of the authors and do not necessarily represent those of their affiliated organizations, or those of the publisher, the editors and the reviewers. Any product that may be evaluated in this article, or claim that may be made by its manufacturer, is not guaranteed or endorsed by the publisher.
